# Production in *Pichia pastoris* of complementary protein-based polymers with heterodimer-forming WW and PPxY domains

**DOI:** 10.1186/s12934-016-0498-3

**Published:** 2016-06-10

**Authors:** Natalia E. Domeradzka, Marc W. T. Werten, Renko de Vries, Frits A. de Wolf

**Affiliations:** Wageningen UR Food and Biobased Research, Bornse Weilanden 9, 6708 WG Wageningen, The Netherlands; Physical Chemistry and Soft Matter, Wageningen University, Stippeneng 4, 6708 WE Wageningen, The Netherlands

**Keywords:** Heterodimerization, O-glycosylation, Phosphorylation, *Pichia pastoris*, PPxY domain, Protein-based polymers, Protein coupling, WW domain

## Abstract

**Background:**

Specific coupling of de novo designed recombinant protein polymers for the construction of precisely structured nanomaterials is of interest for applications in biomedicine, pharmaceutics and diagnostics. An attractive coupling strategy is to incorporate specifically interacting peptides into the genetic design of the protein polymers. An example of such interaction is the binding of particular proline-rich ligands by so-called WW-domains. In this study, we investigated whether these domains can be produced in the yeast *Pichia pastoris* as part of otherwise non-interacting protein polymers, and whether they bring about polymer coupling upon mixing.

**Results:**

We constructed two variants of a highly hydrophilic protein-based polymer that differ only in their C-terminal extensions. One carries a C-terminal WW domain, and the other a C-terminal proline-rich ligand (PPxY). Both polymers were produced in *P.pastoris* with a purified protein yield of more than 2 g L^−1^ of cell-free broth. The proline-rich module was found to be O-glycosylated, and uncommonly a large portion of the attached oligosaccharides was phosphorylated. Glycosylation was overcome by introducing a Ser → Ala mutation in the PPxY peptide. Tryptophan fluorescence monitored during titration of the polymer containing the WW domain with either the glycosylated or nonglycosylated PPxY-containing polymer revealed binding. The complementary polymers associated with a K_d_ of ~3 µM, regardless of glycosylation state of the PPxY domain. Binding was confirmed by isothermal titration calorimetry, with a K_d_ of ~9 µM.

**Conclusions:**

This article presents a blueprint for the production in *P. pastoris* of protein polymers that can be coupled using the noncovalent interaction between WW domains and proline-rich ligands. The availability of this highly specific coupling tool will hereafter allow us to construct various supramolecular structures and biomaterials.

**Electronic supplementary material:**

The online version of this article (doi:10.1186/s12934-016-0498-3) contains supplementary material, which is available to authorized users.

## Background

Protein-based block copolymers (or protein polymers, for short) are de novo designed polypeptides that consist of different blocks, where each of the blocks adopts a specific conformation and fulfills a specific function [1]. Self-assembling protein polymers are being intensively explored for use as biomaterials for drug encapsulation, controlled drug release, tissue engineering, tissue augmentation, and biosensors [2–8]. Protein polymers are produced via recombinant DNA technology, and thus, aside from possible problems with the biological production, in principle have a defined sequence, mass, and chemical composition. They are biodegradable, often biocompatible, and easy to functionalize by inclusion of bioactive sequences in the genetic design [1, 7].

In early work, the sequences of protein polymers were typically inspired by naturally occurring self-assembling structural proteins such as silk, elastin, collagen, and resilin, with inherent attractive properties as soft materials [9, 10]. The repertoire of useful motifs for self-assembly has been expanded over the years with sequences designed or modified using molecular modelling. Several self-assembling modules can be combined into a multifunctional polypeptide block copolymer that can spontaneously self-organize via non-covalent interactions into nanostructures such as micelles, fibrils, or hydrogels [11–13]. However, peptide blocks that can facilitate the assembly of supramolecular structures with particularly high precision are still highly sought after in biomaterial science [14–16].

Our group has successfully produced several protein polymers at high yield using the yeast *Pichia pastoris* [17–23]. Many of these are block copolymers that self-organize into stimulus-responsive supramolecular structures. We currently aim to expand our library of functional protein modules with blocks that allow highly precise heterodimerization, in an effort to gain more control over the self-assembly of supramolecular structures. A pair of block copolymers fitted with complementary interacting modules could then self-assemble into higher order structures upon mere mixing of the two [24]. Besides providing crosslinks in self-assembled structures, heterodimerizing modules can also be used for incorporation of functionalities such as growth factors, antimicrobial peptides, and cell-adhesive peptides [25].

The so-called WW domain is found in various natural proteins and binds particular proline-rich peptides with a high degree of specificity [26]. As its name suggests, the amino acid sequence of the WW domain contains two highly conserved tryptophans. The domain consists of a slightly bent three-stranded antiparallel β-sheet, the concave side of which forms a binding pocket for the proline-rich ligand [27]. Wong Po Foo et al. successfully used the interaction between two different WW domains (CC43 and Nedd4.3) and a so-called group I proline-rich peptide (PPxY) to generate two-component hydrogels [28]. Here, we investigate the separate incorporation of this same PPxY peptide derived from p53-binding protein-2 [29] and another WW domain (WWP1-1; one of three WW domains present in the human ubiquitin ligase homolog WWP1 [29]) at the C-terminus of the ${\mathbf{C}}_{{\mathbf{4}}}^{{\mathbf{P}}}$ protein polymer previously developed by us [18]. The ${\mathbf{C}}_{{\mathbf{4}}}^{{\mathbf{P}}}$ polymer (formerly referred to as ‘P4’) consists of four identical copies of a 99 amino acid long, highly hydrophilic random coil polypeptide [18]. We report the high-yield secretory production of these polymers in *Pichia pastoris* and their characterization. Undesired O-glycosylation of the PPxY peptide was overcome, and the polymers interacted as intended. We have thus expanded our toolkit towards the creation of well-defined supramolecular topologies for new biomaterials.

## Results and discussion

### Protein production and purification

The WW and PPxY domains are referred to hereafter as **D**^**WW**^ and **D**^**PPxY**^, respectively (D for Dimerization). For amino acid sequences see Table 1. The domains were cloned at the 3’ end of the gene encoding the previously reported ${\mathbf{C}}_{{\mathbf{4}}}^{{\mathbf{P}}}$ protein polymer [18]. The encoded proteins ${\mathbf{C}}_{{\mathbf{4}}}^{{\mathbf{P}}} - {\mathbf{D}}^{{{\mathbf{WW}}}}$ and ${\mathbf{C}}_{{\mathbf{4}}}^{{\mathbf{P}}} - {\mathbf{D}}^{{{\mathbf{PPxY}}}}$ were produced in secretory fashion using genetically modified *P. pastoris,* grown in methanol fed-batch mode. Proteins were purified from the cell-free broth by differential ammonium sulfate precipitation, and subsequently dialyzed and lyophilized. Previous studies showed that ammonium sulfate precipitation of protein polymers containing the ${\mathbf{C}}_{{\mathbf{4}}}^{{\mathbf{P}}}$ block typically results in a purity of ~99 % at the protein level [19, 30]. The gravimetrically determined yields, expressed in g per L of cell-free broth, were 2.2 g L^−1^ for ${\mathbf{C}}_{{\mathbf{4}}}^{{\mathbf{P}}} - {\mathbf{D}}^{{{\mathbf{WW}}}}$, and 2.3 g L^−1^ for ${\mathbf{C}}_{{\mathbf{4}}}^{{\mathbf{P}}} - {\mathbf{D}}^{{{\mathbf{PPxY}}}}$. Although several reports describe the production of proteins with WW domains and proline-rich ligands in *Escherichia coli*, yields have not been reported [28, 31, 32]. To our knowledge, production of WW and PPxY domains in *P. pastoris* has not been reported before. The non-optimized yields are in the same g L^−1^ range as for ${\mathbf{C}}_{{\mathbf{4}}}^{{\mathbf{P}}}$ [18], showing that the **D**^**WW**^ and **D**^**PPxY**^ modules are not a significant bottle-neck. This offers good prospects towards their further use in the construction of complex supramolecular architectures and biomaterials.

**Table 1:** Amino acid sequences of the C-terminal **D** ^**ww**^ **, D** ^**PPxY**^ and **D** ^PPxY*^ modules

Module	Amino acid sequence
D^WW^	LPSGWEQRKDPHGRTYYVDHNTRTTTWERPQPLPPGA
D^PPxY^	EYPPYPPPPYPSG
D^PPxY*^	EYPPYPPPPYPAG

All sequences end C-terminally in the cloning-derived amino acid sequence PAGG (not indicated)

### Protein characterization

**Fig. 1:**
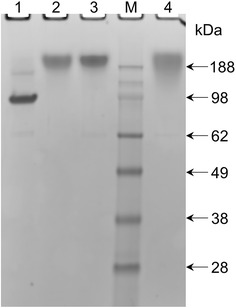
SDS-PAGE analysis of the purified protein polymers. *Lane 1*, ${\mathbf{C}}_{{\mathbf{4}}}^{{\mathbf{P}}} - {\mathbf{D}}^{{{\mathbf{WW}}}}$; *lane 2*, ${\mathbf{C}}_{{\mathbf{4}}}^{{\mathbf{P}}} - {\mathbf{D}}^{{{\mathbf{PPxY}}}}$; *lane 3*, ${\mathbf{C}}_{{\mathbf{4}}}^{{\mathbf{P}}} - {\mathbf{D}}^{{{\mathbf{PPxY}} ^* }}$; *lane M*, protein molecular weight marker; *lane 4*, control protein ${\mathbf{C}}_{{\mathbf{4}}}^{{\mathbf{P}}}$. Samples were loaded at 13 µg of protein per lane

${\mathbf{C}}_{{\mathbf{4}}}^{{\mathbf{P}}} - {\mathbf{D}}^{{{\mathbf{WW}}}}$ and ${\mathbf{C}}_{{\mathbf{4}}}^{{\mathbf{P}}} - {\mathbf{D}}^{{{\mathbf{PPxY}}}}$ were characterized by SDS–PAGE (Fig. 1). Based on the well-established aberrant migration behavior of the ${\mathbf{C}}_{{\mathbf{4}}}^{{\mathbf{P}}}$ block in SDS-PAGE, the proteins were expected to migrate much more slowly in SDS-PAGE than would be expected on the basis of their theoretical molecular weights of ~41 and ~39 kDa, respectively [18, 19, 23, 30]. This is due to the highly hydrophilic character and consequent low SDS-binding capacity of the ${\mathbf{C}}_{{\mathbf{4}}}^{{\mathbf{P}}}$ block [18]. The 13-residue **D**^**PPxY**^ block resembles the proline-rich nature of the ${\mathbf{C}}_{{\mathbf{4}}}^{{\mathbf{P}}}$ block, and as such is not expected to much affect the mobility of the protein in SDS-PAGE relative to that of ${\mathbf{C}}_{{\mathbf{4}}}^{{\mathbf{P}}}$ alone. On the other hand, the 37-residue hydrophobic **D**^**WW**^ block likely will, because attachment to ${\mathbf{C}}_{{\mathbf{4}}}^{{\mathbf{P}}}$ of peptides that improve SDS binding is known to increase the mobility of the polymer [23, 30]. Indeed, the proteins migrated accordingly in SDS-PAGE (Fig. 1): ${\mathbf{C}}_{{\mathbf{4}}}^{{\mathbf{P}}} - {\mathbf{D}}^{{{\mathbf{PPxY}}}}$ migrates as slowly as the control ${\mathbf{C}}_{{\mathbf{4}}}^{{\mathbf{P}}}$ protein, and ${\mathbf{C}}_{{\mathbf{4}}}^{{\mathbf{P}}} - {\mathbf{D}}^{{{\mathbf{WW}}}}$ migrates faster than ${\mathbf{C}}_{{\mathbf{4}}}^{{\mathbf{P}}}$. Although SDS-PAGE is not informative about the molecular mass of the polymers, it does show the proteins are relatively pure and intact.

**Fig. 2:**
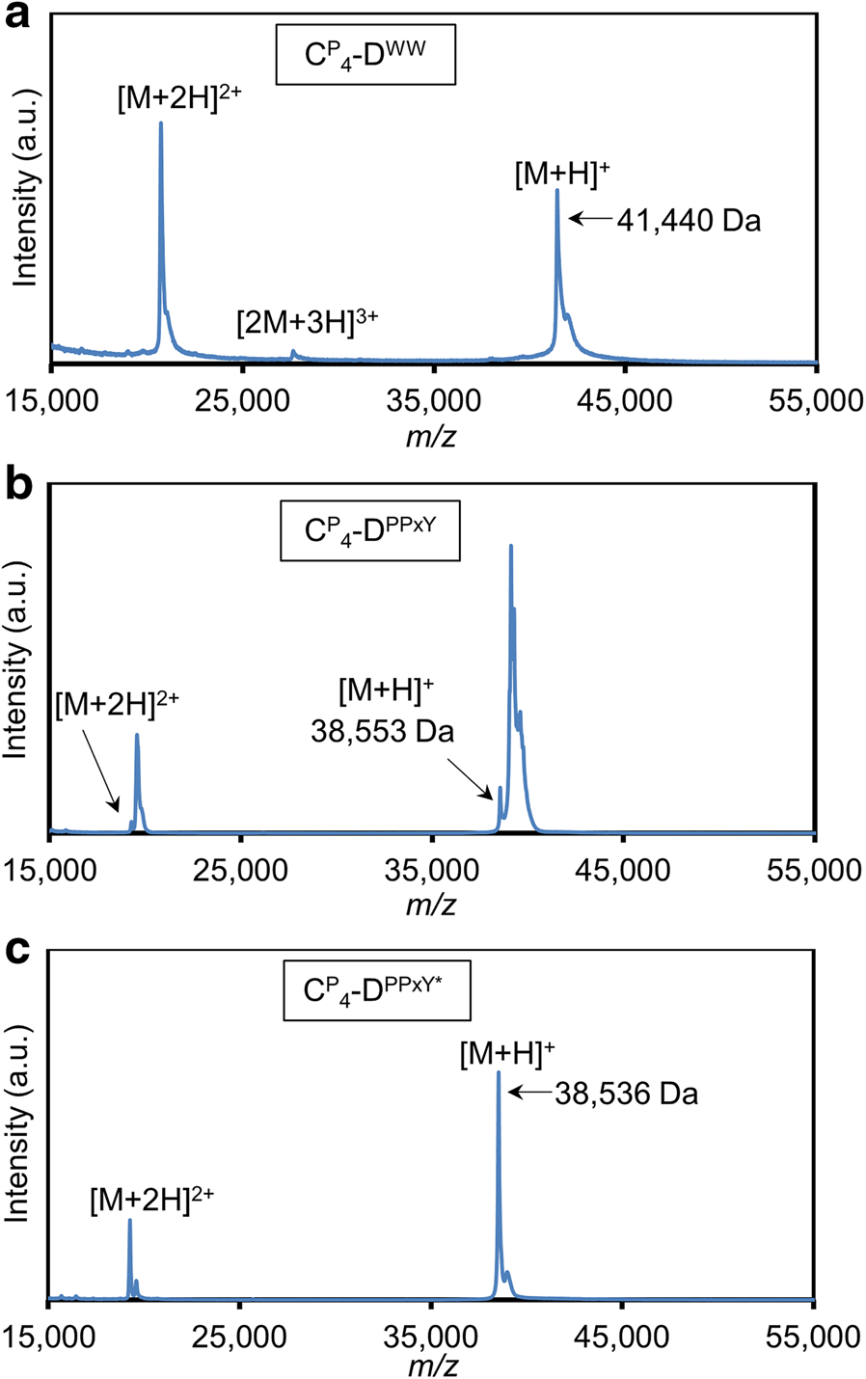
MALDI-TOF analysis of the purified protein polymers. ${\mathbf{C}}_{{\mathbf{4}}}^{{\mathbf{P}}} - {\mathbf{D}}^{{{\mathbf{WW}}}}$ (**a**), ${\mathbf{C}}_{{\mathbf{4}}}^{{\mathbf{P}}} - {\mathbf{D}}^{{{\mathbf{PPxY}}}}$ (**b**), ${\mathbf{C}}_{{\mathbf{4}}}^{{\mathbf{P}}} - {\mathbf{D}}^{{{\mathbf{PPxY}} ^* }}$ (**c**)

**Fig. 3:**
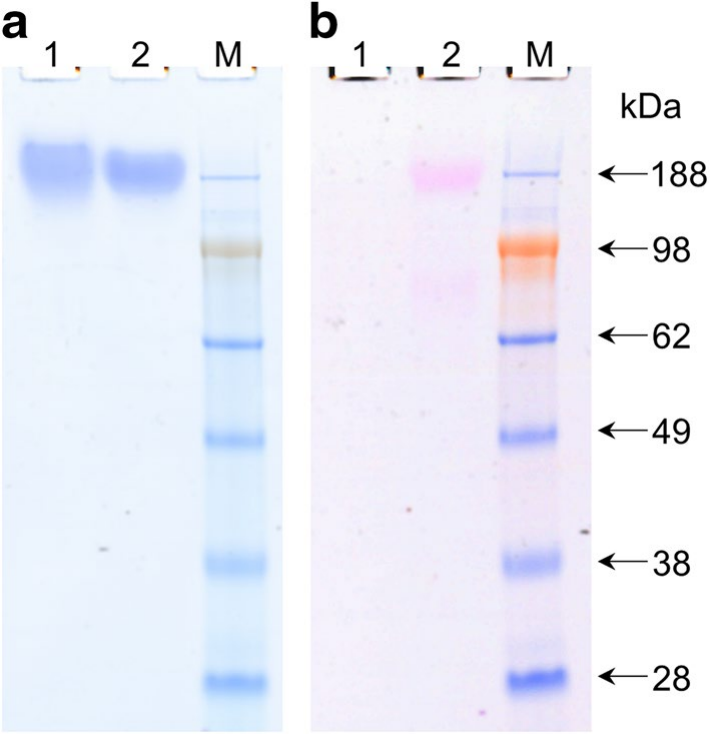
Detection of glycosylation by SDS-PAGE. *Lane 1*, ${\mathbf{C}}_{{\mathbf{4}}}^{{\mathbf{P}}}$ control; *lane 2*, ${\mathbf{C}}_{{\mathbf{4}}}^{{\mathbf{P}}} - {\mathbf{D}}^{{{\mathbf{PPxY}}}}$; *lane M*: protein molecular weight marker. Stained with Coomassie Brilliant Blue (**a**). Stained using Periodic acid-Schiff staining (**b**). Equal amounts of protein were loaded for all samples

The molecular weight distribution of purified ${\mathbf{C}}_{{\mathbf{4}}}^{{\mathbf{P}}} - {\mathbf{D}}^{{{\mathbf{WW}}}}$ and ${\mathbf{C}}_{{\mathbf{4}}}^{{\mathbf{P}}} - {\mathbf{D}}^{{{\mathbf{PPxY}}}}$ was further investigated by MALDI-TOF mass spectrometry. A peak at *m*/*z* 41,440 was observed in the spectrum of ${\mathbf{C}}_{{\mathbf{4}}}^{{\mathbf{P}}} - {\mathbf{D}}^{{{\mathbf{WW}}}}$ (Fig. 2a). This peak corresponds, within experimental error, to the expected molecular weight of the intact protein (41,437 Da). The small shoulder represents a minor fraction of the molecules with an N-terminal (Glu-Ala)_2_ extension. Such extensions commonly occur in *P. pastoris* due to incomplete processing of the α-factor prepro secretory signal [17]. The MALDI-TOF analysis confirms the conclusion from SDS-PAGE that the ${\mathbf{C}}_{{\mathbf{4}}}^{{\mathbf{P}}} - {\mathbf{D}}^{{{\mathbf{WW}}}}$ protein is pure and intact. The MALDI-TOF spectrum for ${\mathbf{C}}_{{\mathbf{4}}}^{{\mathbf{P}}} - {\mathbf{D}}^{{{\mathbf{PPxY}}}}$ (Fig. 2b), however, showed several peaks. The minor low mass peak at *m*/*z* 38,553 is in accordance with the expected molecular weight of the intact protein (38,552 Da). It seems likely that the other peaks of higher molecular mass represent glycosylated species. Indeed, SDS-PAGE followed by Periodic acid-Schiff staining [33] confirmed that ${\mathbf{C}}_{{\mathbf{4}}}^{{\mathbf{P}}} - {\mathbf{D}}^{{{\mathbf{PPxY}}}}$ is glycosylated (Fig. 3). Although the sequence of ${\mathbf{C}}_{{\mathbf{4}}}^{{\mathbf{P}}} - {\mathbf{D}}^{{{\mathbf{PPxY}}}}$ contains no N-x-[ST] N-glycosylation motifs, *P. pastoris* is also capable of O-glycosylation [34]. Because ${\mathbf{C}}_{{\mathbf{4}}}^{{\mathbf{P}}}$, as a separate protein, is known to be nonglycosylated [18] (see also Fig. 3) most likely the single Ser residue in the added **D**^**PPxY**^ block has been modified with O-glycans.

### Construction of a Ser12 → Ala PPxY mutant

Glyosylation of therapeutic proteins by *P. pastoris* may cause adverse immune responses [35]. Also from a nanomaterials point of view, the polydispersity resulting from glycosylation is undesirable, and desired interactions may well be hindered by the presence of oligosaccharides. To abolish the observed glycosylation of ${\mathbf{C}}_{{\mathbf{4}}}^{{\mathbf{P}}} - {\mathbf{D}}^{{{\mathbf{PPxY}}}}$, we constructed a variant with a Ser12→Ala mutation in the PPxY module (Table 1). This variant, denoted ${\mathbf{C}}_{{\mathbf{4}}}^{{\mathbf{P}}} - {\mathbf{D}}^{{{\mathbf{PPxY}} ^* }}$, was produced in *P. pastoris* and purified as described above. SDS-PAGE shows a single band (Fig. 1), and the yield of purified protein was 2.5 g L^−1^ of cell-free broth. MALDI-TOF of ${\mathbf{C}}_{{\mathbf{4}}}^{{\mathbf{P}}} - {\mathbf{D}}^{{{\mathbf{PPxY}} ^* }}$ no longer showed the extensive pattern of glycosylated species (Fig. 2c). Instead, a major peak at *m*/*z* 38,536 is seen, matching the expected molecular weight of 38,536 Da. The shoulder represents a minor fraction of the protein not fully processed by *P. pastoris* dipeptidylaminopeptidase, as mentioned above.

The absence of glycosylation in ${\mathbf{C}}_{{\mathbf{4}}}^{{\mathbf{P}}} - {\mathbf{D}}^{{{\mathbf{PPxY}} ^* }}$ shows that indeed Ser12 of the PPxY module in ${\mathbf{C}}_{{\mathbf{4}}}^{{\mathbf{P}}} - {\mathbf{D}}^{{{\mathbf{PPxY}}}}$ was glycosylated. Although there is no known consensus sequence for O-glycosylation, serine/threonine rich sequences appear relatively susceptible, particularly when prolines are in the vicinity of the hydroxyl residues [36]. Although both ${\mathbf{C}}_{{\mathbf{4}}}^{{\mathbf{P}}}$ and **D**^**PPxY**^ are rich in proline and serine, interestingly, all serines in ${\mathbf{C}}_{{\mathbf{4}}}^{{\mathbf{P}}}$ are followed by proline, while in **D**^**PPxY**^ the single serine is preceded by proline. The observed exclusive glycosylation of serine in the PPxY module seems to agree with the reported enhancement of mannosyl transfer in *S. cerevisiae* for peptide substrates with proline N-terminal to the hydroxyl amino acid [37], and with its inhibition when proline is the C-terminal neighbor [38]. However, this should not be taken to imply that such simple motifs are sufficient to determine O-glycosylation.

### Limited glycan characterization

The fact that exclusively Ser12 in the PPxY module was glycosylated allows a direct interpretation of the mass spectrum of the glycosylated protein in terms of the oligosaccharide composition of the population of attached glycans. Although relatively few studies have reported O-glycosylation in *P. pastoris*, it is clear that O-glycans in this host usually consist of up to five mannose units [34], with dimers and trimers being the most abundant species [39, 40]. However, longer glycan chains of up to nine mannose residues have been described [41], as well as a phosphorylated Man_6_ O-glycan [40]. The glycosidic links between the mannose units are mostly in α1-2 arrangement [39, 40], although also α1-3 and α1-6 links have been reported [41, 42], as well as ß1-2 links [40].

**Fig. 4:**
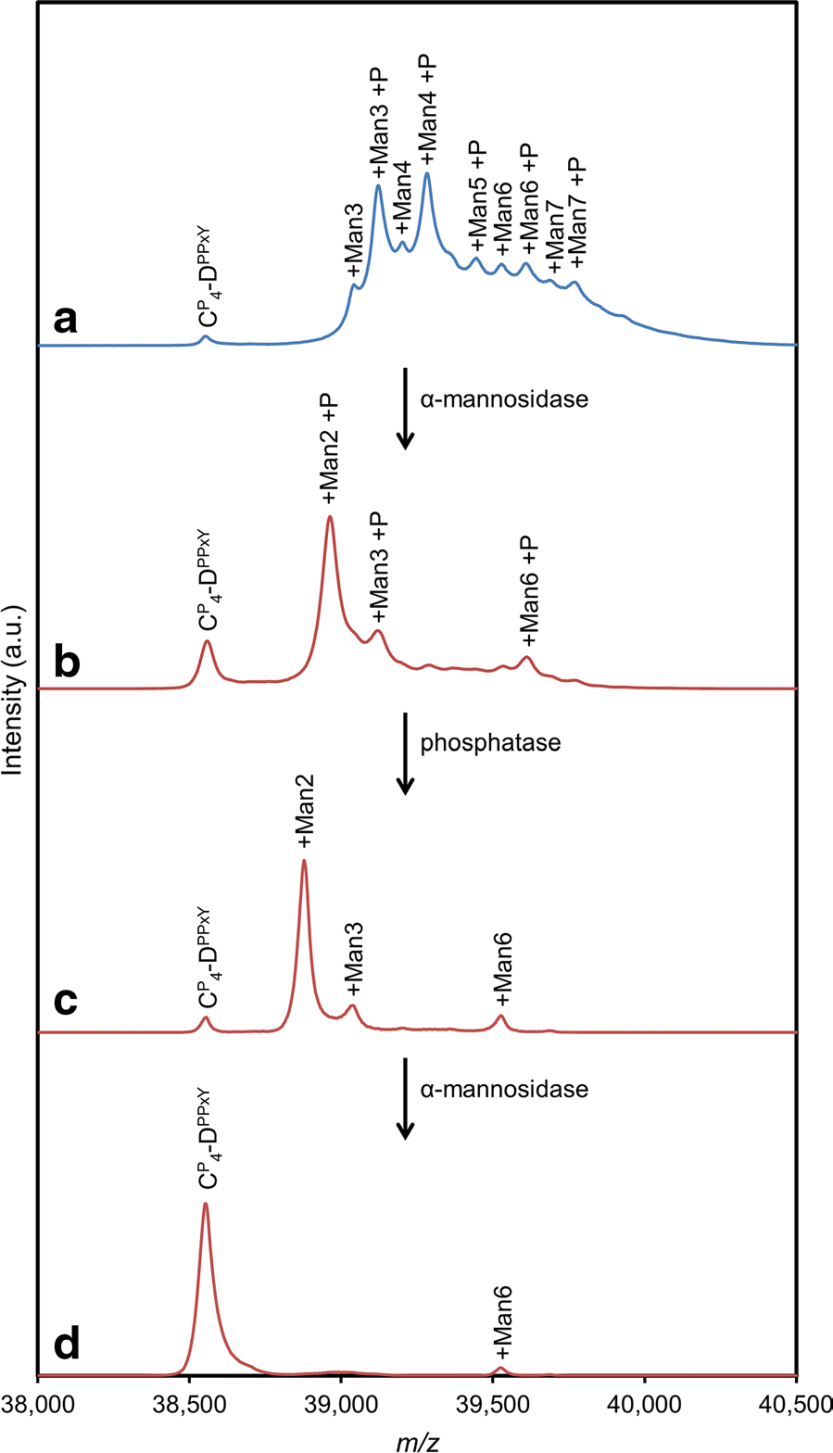
MALDI-TOF of ${\mathbf{C}}_{{\mathbf{4}}}^{{\mathbf{P}}} - {\mathbf{D}}^{{{\mathbf{PPxY}}}}$ treated with α(1-2,1-3,1-6) mannosidase and phosphatase. Untreated ${\mathbf{C}}_{{\mathbf{4}}}^{{\mathbf{P}}} - {\mathbf{D}}^{{{\mathbf{PPxY}}}}$ (**a**). The protein was digested with α-mannosidase (**b**), then with phosphatase (**c**), and finally digested again with α-mannosidase (**d**). Enzymes were thermally inactivated between consecutive digestions. See Table 2 for observed *m*/*z* values and theoretical masses of the indicated glycoforms

For closely studying the glycan mass distribution, the MALDI-TOF analysis of ${\mathbf{C}}_{{\mathbf{4}}}^{{\mathbf{P}}} - {\mathbf{D}}^{{{\mathbf{PPxY}}}}$ was repeated with optimal settings for the *m*/*z* range of the relevant [M + H]^+^ ions (Fig. 4a). Table 2 provides an interpretation of the observed peaks, all of which can be explained in terms of mannose units (+162 Da mass shift) and phosphorylation (+80 Da mass shift). Applying Occam’s razor, we assumed maximally one phosphate per glycan structure.

**Table 2:** Masses observed in MALDI-TOF and tentative glycan structures

	*m*/*z* ^a^	Additional mass (Da)^b^	Tentative glycan structure^c^	Theoretical mass of tentative glycan structure (Da)^d^
${\mathbf{C}}_{{\mathbf{4}}}^{{\mathbf{P}}} - {\mathbf{D}}^{{{\mathbf{PPxY}}}}$	38,553	–	–	–
	39,042	489	Man_3_	486
	39,122	569	Man_3_ + P	566
	39,202	649	Man_4_	649
	39,283	730	Man_4_ + P	729
	39,444	891	Man_5_ + P	891
	39,529	976	Man_6_	973
	39,609	1056	Man_6_ + P	1053
	39,688	1135	Man_7_	1135
	39,767	1214	Man_7_ + P	1215
${\mathbf{C}}_{{\mathbf{4}}}^{{\mathbf{P}}} - {\mathbf{D}}^{{{\mathbf{PPxY}}}}$ digested with α-mannosidase	38,558	–	–	–
	38,963	405	Man_2_ + P	404
	39,121	563	Man_3_ + P	566
	39,611	1053	Man_6_ + P	1053
${\mathbf{C}}_{{\mathbf{4}}}^{{\mathbf{P}}} - {\mathbf{D}}^{{{\mathbf{PPxY}}}}$ digested with (1) α-mannosidase, and (2) phosphatase^e^	38,553	–	–	–
	38,878	325	Man_2_	324
	39,039	486	Man_3_	486
	39,526	973	Man_6_	973
${\mathbf{C}}_{{\mathbf{4}}}^{{\mathbf{P}}} - {\mathbf{D}}^{{{\mathbf{PPxY}}}}$ digested with (1) α-mannosidase, (2) phosphatase, and (3) α-mannosidase^e^	38,553	–	–	–
	39,526	973	Man_6_	973

^a^See Fig. 4 (only true peaks are listed; minor inflections in the spectra are ignored)

^b^Relative to the *m*/*z* value corresponding to the nonglycosylated protein in the same mass spectrum

^c^Assuming mannose (Man) units only, and maximally one phosphate (P) per glycan

^d^Theoretical glycoform masses calculated using 162.14 Da for Man and 79.98 Da for P

^e^Enzymes were thermally inactivated between consecutive digestions

Hypothetically, the ${\mathbf{C}}_{{\mathbf{4}}}^{{\mathbf{P}}} - {\mathbf{D}}^{{{\mathbf{PPxY}}}}$ protein itself may have been phosphorylated, rather than the oligosaccharides attached to it. Because a nonglycosylated peak shifted by +80 Da is not observed in the MALDI-TOF spectrum of ${\mathbf{C}}_{{\mathbf{4}}}^{{\mathbf{P}}} - {\mathbf{D}}^{{{\mathbf{PPxY}}}}$, and because both ${\mathbf{C}}_{{\mathbf{4}}}^{{\mathbf{P}}}$ [18] and the nonglycosylated ${\mathbf{C}}_{{\mathbf{4}}}^{{\mathbf{P}}} - {\mathbf{D}}^{{{\mathbf{PPxY}}^ * }}$ are not phosphorylated, such hydroxyl amino acid phosphorylation would need to have occurred specifically in the glycosylated fraction of the ${\mathbf{C}}_{{\mathbf{4}}}^{{\mathbf{P}}} - {\mathbf{D}}^{{{\mathbf{PPxY}}}}$ molecules. This unlikely notion can be excluded because treatment with alkaline phosphatase did not result in significant changes to the MALDI-TOF spectrum (Additional file 1: Fig. S1). This finding furthermore indicates that the phosphorylated oligosaccharides do not contain phosphomonoesters, but rather contain phosphorylated Man in diester linkage, in agreement with the findings by Trimble et al. for the above-mentioned phosphorylated Man_6_ O-glycan [40].

When ${\mathbf{C}}_{{\mathbf{4}}}^{{\mathbf{P}}} - {\mathbf{D}}^{{{\mathbf{PPxY}}}}$ was treated with the exoglycosidase jack bean α(1-2,1-3,1-6) mannosidase, a clearly altered mass distribution was obtained in MALDI-TOF (Fig. 4b; Table 2). Repeated digestions using increasing amounts of enzyme and incubation time all resulted in similar spectra (not shown), suggesting that the glycan structure is partially resistant to the α(1-2,1-3,1-6) mannosidase. The observed phosphorylation provides a likely explanation. Nonphosphorylated species were hardly detectable after digestion with jack bean mannosidase (Fig. 4b; Table 2). Furthermore, a peak corresponding to Man_2_ + P is absent in the undigested sample, but appears upon α-mannosidase digestion at the apparent expense of Man_3_ + P and larger phosphorylated forms. Although jack bean α-mannosidase can trim terminal mannoses linked to phosphate, it cannot thereafter proceed further [43–45]. Thus, the digestion likely halted upon generation of the phosphorylated species observed in Fig. 4b (Man_2_ + P, Man_3_ + P, and Man_6_ + P). Treatment of the mannosidase-digested sample with alkaline phosphatase resulted in a shift by −80 Da for these three phosphorylated species (Fig. 4c; Table 2), confirming that they were present as phosphodiesters prior to α-mannosidase digestion, and that indeed, as assumed above, each glycoform contains only one phosphate. Moreover, dephosphorylation rendered the remaining glycan structures susceptible to further digestion by α-mannosidase (Fig. 4d; Table 2). Possibly, the minor amount of remaining Man_6_ is capped with a resistant β1-2 Man disaccharide, as has been described for *P. pastoris* [40].

A detailed study of O-glycans chemically released from ${\mathbf{C}}_{{\mathbf{4}}}^{{\mathbf{P}}} - {\mathbf{D}}^{{{\mathbf{PPxY}}}}$ would be needed to determine their precise structure with certainty, but is beyond the scope of this work. Nonetheless, it is clear that the heterogeneous mixture of glycans attached to Ser12 in the PPxY module contains phosphorylated species in diester linkage. Similarly phosphorylated O-glycans have been described for several proteins in *S. cerevisiae* [44–46]. To our knowledge, the present study represents the first confirmation of the occurrence of phosphorylated O-glycans in *P. pastoris* as reported by Trimble et al. [40]. Interestingly, the phosphorylated Man_6_ previously reported was described as only a minor component [40], whereas most of the oligosaccharides on **C**_**4**_**-D**^**PPxY**^ are phosphorylated.

### Binding of proline-rich ligands by the WW domain

Because tryptophans are exclusively present in the WW domain, tryptophan fluorescence can be used to monitor binding of ${\mathbf{C}}_{{\mathbf{4}}}^{{\mathbf{P}}} - {\mathbf{D}}^{{{\mathbf{WW}}}}$ to its proline-rich ligands. Upon binding, the local environment of the tryptophans becomes more hydrophobic, causing a blue-shift of the emission maximum and increased fluorescence. Tryptophan fluorescence of a fixed amount of ${\mathbf{C}}_{{\mathbf{4}}}^{{\mathbf{P}}} - {\mathbf{D}}^{{{\mathbf{WW}}}}$ (10 µM) was thus followed during titration with a concentrated stock solution of either ${\mathbf{C}}_{{\mathbf{4}}}^{{\mathbf{P}}} - {\mathbf{D}}^{{{\mathbf{PPxY}}}}$ or ${\mathbf{C}}_{{\mathbf{4}}}^{{\mathbf{P}}} - {\mathbf{D}}^{{{\mathbf{PPxY}} ^* }}$.

**Fig. 5:**
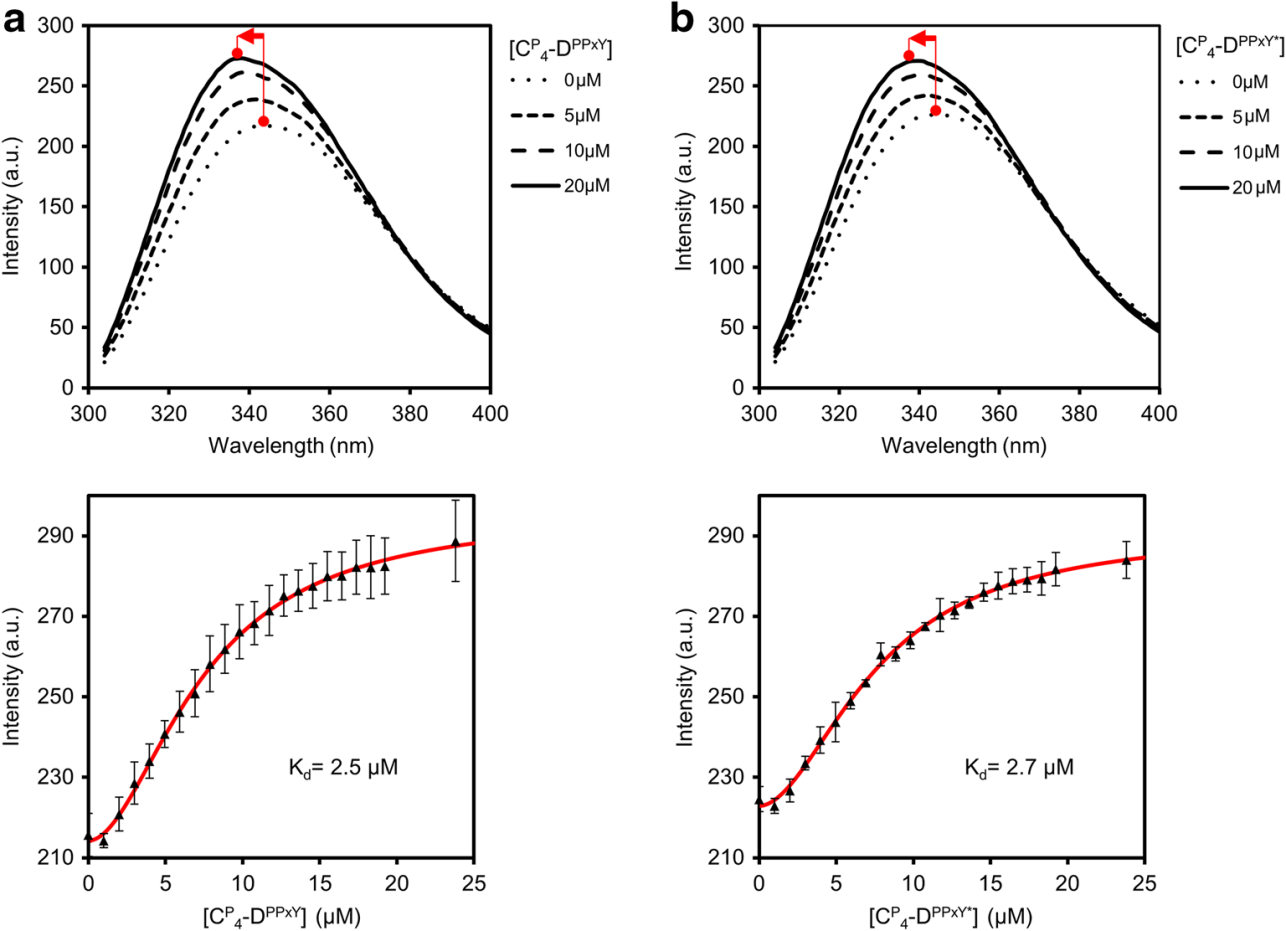
Steady-state tryptophan fluorescence of ${\mathbf{C}}_{{\mathbf{4}}}^{{\mathbf{P}}} - {\mathbf{D}}^{{{\mathbf{WW}}}}$ upon titration with ${\mathbf{C}}_{{\mathbf{4}}}^{{\mathbf{P}}} - {\mathbf{D}}^{{{\mathbf{PPxY}}}}$ (**a**) and ${\mathbf{C}}_{{\mathbf{4}}}^{{\mathbf{P}}} - {\mathbf{D}}^{{{\mathbf{PPxY}} ^* }}$ (**b**). The *top graphs* present tryptophan fluorescence spectra obtained for ${\mathbf{C}}_{{\mathbf{4}}}^{{\mathbf{P}}} - {\mathbf{D}}^{{{\mathbf{WW}}}}$ upon addition of different concentrations of ${\mathbf{C}}_{{\mathbf{4}}}^{{\mathbf{P}}} - {\mathbf{D}}^{{{\mathbf{PPxY}}}}$ and ${\mathbf{C}}_{{\mathbf{4}}}^{{\mathbf{P}}} - {\mathbf{D}}^{{{\mathbf{PPxY}} ^* }}$. The *bottom graphs* show titration of ${\mathbf{C}}_{{\mathbf{4}}}^{{\mathbf{P}}} - {\mathbf{D}}^{{{\mathbf{WW}}}}$ with ${\mathbf{C}}_{{\mathbf{4}}}^{{\mathbf{P}}} - {\mathbf{D}}^{{{\mathbf{PPxY}}}}$ and ${\mathbf{C}}_{{\mathbf{4}}}^{{\mathbf{P}}} - {\mathbf{D}}^{{{\mathbf{PPxY}} ^* }}$, where fluorescence was monitored at 340 nm. *Error bars* represent s.d. (*n* = 3)

An excitation wavelength of 295 nm was used to prevent excitation of tyrosines [47], which are present in the PPxY domain. With increasing concentration of the ligand, the maximum emission wavelength of ${\mathbf{C}}_{{\mathbf{4}}}^{{\mathbf{P}}} - {\mathbf{D}}^{{{\mathbf{WW}}}}$ decreased, indicating the transition of at least one tryptophan of ${\mathbf{C}}_{{\mathbf{4}}}^{{\mathbf{P}}} - {\mathbf{D}}^{{{\mathbf{WW}}}}$ from a solvent-exposed to a more hydrophobic environment (Fig. 5). As expected, this was accompanied by an increased fluorescence quantum yield. From the titration plots in Fig. 5, a K_d_ of 2.5 µM was calculated by non-linear regression for ${\mathbf{C}}_{{\mathbf{4}}}^{{\mathbf{P}}} - {\mathbf{D}}^{{{\mathbf{PPxY}}}}$, and a K_d_ of 2.7 µM for ${\mathbf{C}}_{{\mathbf{4}}}^{{\mathbf{P}}} - {\mathbf{D}}^{{{\mathbf{PPxY}} ^* }}$. Apparently, the WW domain binds equally well to glycosylated and nonglycosylated ${\mathbf{C}}_{{\mathbf{4}}}^{{\mathbf{P}}} - {\mathbf{D}}^{{{\mathbf{PPxY}}}}$.

**Fig. 6:**
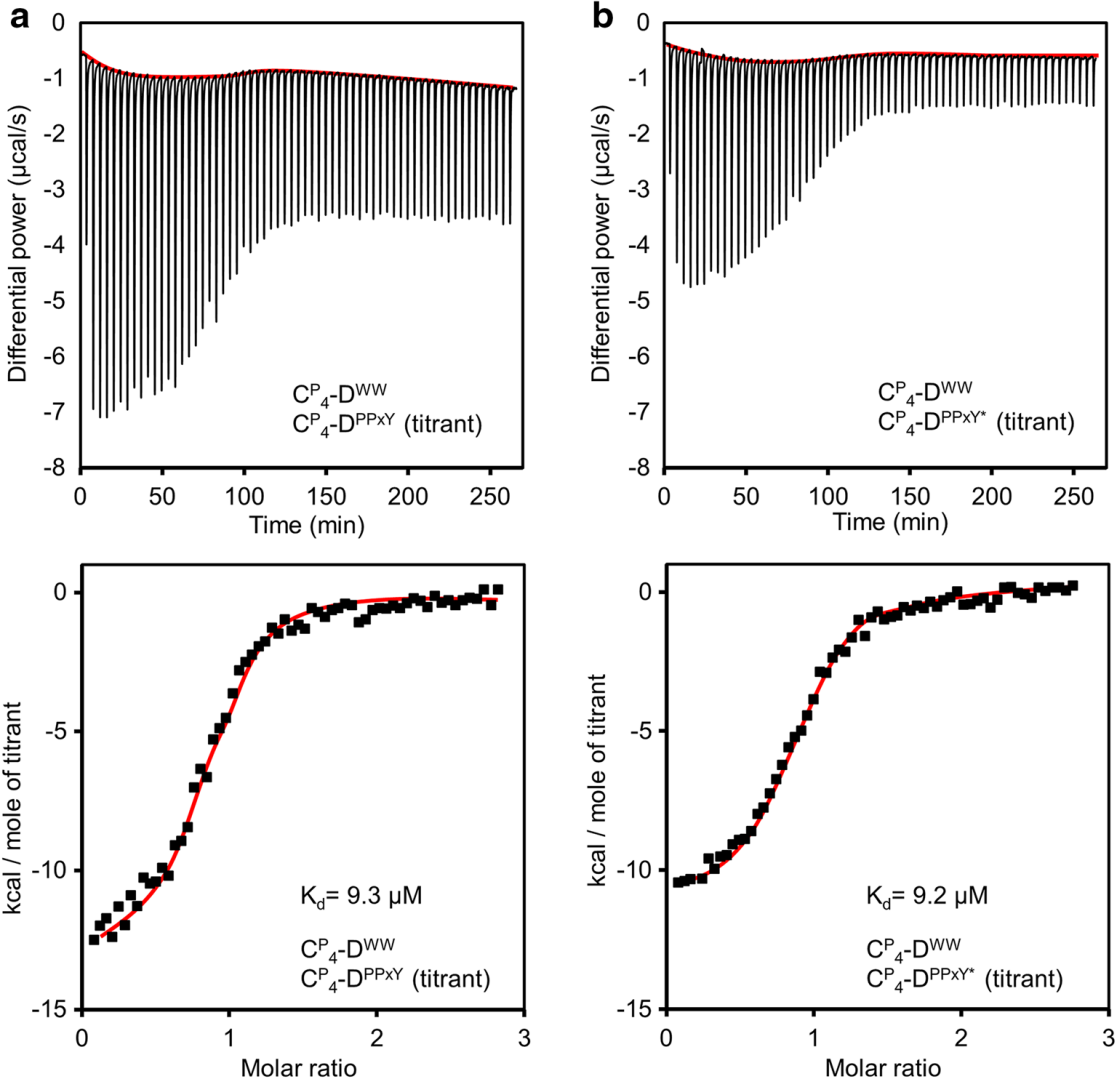
Binding study using isothermal titration calorimetry. ${\mathbf{C}}_{{\mathbf{4}}}^{{\mathbf{P}}} - {\mathbf{D}}^{{{\mathbf{PPxY}}}}$ titrant (**a**), ${\mathbf{C}}_{{\mathbf{4}}}^{{\mathbf{P}}} - {\mathbf{D}}^{{{\mathbf{PPxY}} ^* }}$ titrant (**b**). The *top graphs* show the heat response upon titration of ${\mathbf{C}}_{{\mathbf{4}}}^{{\mathbf{P}}} - {\mathbf{D}}^{{{\mathbf{WW}}}}$ with the proline-rich ligands. *Bottom graphs* represent the integrated peak areas per mole of ligand

Binding affinities were further established using isothermal titration calorimetry. A fixed amount of ${\mathbf{C}}_{{\mathbf{4}}}^{{\mathbf{P}}} - {\mathbf{D}}^{{{\mathbf{WW}}}}$ (200 µM) was titrated with concentrated ligand stock solution (Fig. 6). Control experiments where buffer was titrated with ligand, resulted in a relatively small and constant heat of dilution (Additional file 1: Fig S2A, B). Also ${\mathbf{C}}_{{\mathbf{4}}}^{{\mathbf{P}}} - {\mathbf{D}}^{{{\mathbf{WW}}}}$ titrated with control protein ${\mathbf{C}}_{{\mathbf{4}}}^{{\mathbf{P}}}$ showed only heat of dilution (Additional file 1: Fig. S2C). The binding isotherms of both ${\mathbf{C}}_{{\mathbf{4}}}^{{\mathbf{P}}} - {\mathbf{D}}^{{{\mathbf{PPxY}}}}$ and ${\mathbf{C}}_{{\mathbf{4}}}^{{\mathbf{P}}} - {\mathbf{D}}^{{{\mathbf{PPxY}} ^* }}$ showed an immediate decrease in differential power for each consecutive injection (Fig. 6). The K_d_ values derived from the integrated heat plots in Fig. 6 are 9.3 and 9.2 µM for ${\mathbf{C}}_{{\mathbf{4}}}^{{\mathbf{P}}} - {\mathbf{D}}^{{{\mathbf{PPxY}}}}$ and ${\mathbf{C}}_{{\mathbf{4}}}^{{\mathbf{P}}} - {\mathbf{D}}^{{{\mathbf{PPxY}} ^* }}$, respectively. Both values are similar, and in reasonable agreement with the fluorescence spectroscopy results. In general, the K_d_ of various WW-domains and their proline-rich ligands are in the high nM to low µM range [48]. According to Russ et al., the PPxY peptide, also used in our **D**^**PPxY**^ block, binds the CC43 WW domain with K_d_ = 1.7 µM, and the Nedd4.3 WW domain with K_d_ = 11.2 µM [49]. Wong Po Foo et al. used the same couples in the context of protein polymers and found relatively high K_d_ values of 4.6 µM and 62 µM, respectively, for polymers containing *three* PPxY motifs, interacting with polymers containing *three* CC43 or Nedd4.3 domains. The combination of PPxY (p53BP-2) and WWP1-1 used by us was among the best performing pairs tested by Porozzi et al. [29], but to our knowledge no K_d_ values have been published. In our protein polymer context, the K_d_ of this combination (~3 to 9 µM) is in a similar range as the above-mentioned literature values for other WW/PPxY combinations. This range is quite sufficient for various supramolecular systems, and multiple **D** blocks could be introduced into the polymer for applications that require even lower working concentrations. We did attempt to produce the CC43 domain as well, but encountered proteolytic degradation in *P. pastoris* that could not be readily resolved. The stoichiometry (N) determined for both ${\mathbf{C}}_{{\mathbf{4}}}^{{\mathbf{P}}} - {\mathbf{D}}^{{{\mathbf{PPxY}}}}$ and ${\mathbf{C}}_{{\mathbf{4}}}^{{\mathbf{P}}} - {\mathbf{D}}^{{{\mathbf{PPxY}} ^* }}$ is around 0.9, which, given unavoidable inaccuracies in preparing stock solutions from lyophilized proteins, is in good agreement with the expected 1:1 stoichiometry.

## Conclusion

We have shown that polymers containing the WW domain and a proline-rich ligand can be efficiently produced in *Pichia pastoris*. The PPxY module was found to be O-glycosylated, and remarkably a considerable fraction of the oligomannose structures was phosphorylated. O-glycosylation was abolished by changing the serine in the PPxY sequence to alanine. The WW domain effectively bound both the glycosylated and nonglycosylated PPxY modules, with similar binding affinities. This work provides proof-of-concept that otherwise noninteracting protein polymers can be brought together using the specifically interacting WW and PPxY modules. This will allow us to create protein materials with still better controlled structures at the nanoscale, and hence biomedical materials with more precisely defined interactions with living cells.

## Methods

### Construction of expression vectors and strains

The double-stranded gene fragments encoding **D**^**WW**^ and **D**^**PPxY**^ were assembled via overlap extension PCR [50] from the oligonucleotides shown in Additional file 1: Table S1. The gene fragments were digested with *Xho*I/*Eco*RI and cloned into *Xho*I/*Eco*RI-digested vector pMTL23Δ*Bsa*I [51], in order to obtain two vectors pMTL23Δ*Bsa*I-**D**^**WW**^ and pMTL23Δ*Bsa*I-**D**^**PPxY**^. The vector pMTL23-${\mathbf{C}}_{{\mathbf{4}}}^{{\mathbf{P}}}$ contains the sequence encoding ${\mathbf{C}}_{{\mathbf{4}}}^{{\mathbf{P}}}$ (previously referred to as ‘P4’) [18], and was opened at the 3’ end of the ${\mathbf{C}}_{{\mathbf{4}}}^{{\mathbf{P}}}$ gene with *Van*91I/*Eco*RI. The newly prepared constructs pMTL23Δ*Bsa*I-**D**^**WW**^ and pMTL23Δ*Bsa*I-**D**^**PPxY**^ were digested with *Dra*III/*Eco*RI to release inserts **D**^**WW**^ and **D**^**PPxY**^. The inserts were ligated into the opened pMTL23-${\mathbf{C}}_{{\mathbf{4}}}^{{\mathbf{P}}}$ vector, resulting in pMTL23-${\mathbf{C}}_{{\mathbf{4}}}^{{\mathbf{P}}} - {\mathbf{D}}^{{{\mathbf{WW}}}}$ and pMTL23-${\mathbf{C}}_{{\mathbf{4}}}^{{\mathbf{P}}} - {\mathbf{D}}^{{{\mathbf{PPxY}}}}$.

A Ser12 → Ala mutant of the **D**^**PPxY**^ module was prepared by annealing of a pair of largely complementary oligos (Additional file 1: Table S1), and is referred to as **D**^PPxY*^. This double-stranded adapter with *Dra*III/*Eco*RI overhangs was ligated into vector pMTL23-${\mathbf{C}}_{{\mathbf{4}}}^{{\mathbf{P}}}$ previously digested with *Van*91I/*Eco*RI (at the 3’ end of the ${\mathbf{C}}_{{\mathbf{4}}}^{{\mathbf{P}}}$ gene), resulting in pMTL23Δ*Bsa*I-${\mathbf{C}}_{{\mathbf{4}}}^{{\mathbf{P}}} - {\mathbf{D}}^{{{\mathbf{PPxY}} ^* }}$.

The ${\mathbf{C}}_{{\mathbf{4}}}^{{\mathbf{P}}} - {\mathbf{D}}^{{{\mathbf{WW}}}}$, ${\mathbf{C}}_{{\mathbf{4}}}^{{\mathbf{P}}} - {\mathbf{D}}^{{{\mathbf{PPxY}}}}$, and ${\mathbf{C}}_{{\mathbf{4}}}^{{\mathbf{P}}} - {\mathbf{D}}^{{{\mathbf{PPxY}} ^* }}$ inserts were then released with *Xho*I/*Eco*RI and cloned into the likewise-digested *Pichia pastoris* expression vector pPIC9 (ThermoFisher, Bleiswijk, The Netherlands). This resulted in the construction of the vectors: pPIC9-${\mathbf{C}}_{{\mathbf{4}}}^{{\mathbf{P}}} - {\mathbf{D}}^{{{\mathbf{WW}}}}$, pPIC9-${\mathbf{C}}_{{\mathbf{4}}}^{{\mathbf{P}}} - {\mathbf{D}}^{{{\mathbf{PPxY}}}}$ and pPIC9-${\mathbf{C}}_{{\mathbf{4}}}^{{\mathbf{P}}} - {\mathbf{D}}^{{{\mathbf{PPxY}}^* }}$, respectively. The vectors were linearized with *Sal*I to target for integration at the *his*4 locus. Transformation of *P. pastoris* GS115 by electroporation and selection of Mut^+^ transformants were performed as described previously [17].

### Fermentation

The fermentation setup consisted of a 2.5-L Bioflo 3000 stirred-tank bioreactor (New Brunswick Scientific, Nijmegen, The Netherlands) interfaced with BioCommand Software (New Brunswick Scientific, Nijmegen, The Netherlands) and a homebuilt methanol sensor-controller. The fermentations were performed as described previously [19], as follows. A starting volume of 1.25 L minimal basal salts medium [52] was used. The cultures were always inoculated with precultures grown to similar OD_600_. Growth temperature was 30 °C, and the pH was controlled at 3.0 throughout the entire fermentation. The air was supplemented with 20 % (v/v) oxygen during the glycerol fed-batch phase and the methanol fed-batch phase. During the latter protein production phase, lasting two days, methanol levels were kept at 0.2 % (w/v). Wet biomass was typically ~150 g L^−1^ at the end of the glycerol fed-batch phase, and ~500 g L^−1^ at the end of the fermentation. After harvesting, cells were removed from the broth by centrifugation for 20 min. at 15,000×*g* (RT), followed by microfiltration.

### Protein purification

Purification of all protein polymers was done by ammonium sulfate precipitation essentially as described [19], except that heating of the supernatant and acetone precipitation were omitted. Shortly, medium salts were removed by raising the pH of the cell-free broth to 8.0 with sodium hydroxide, followed by 30 min. of centrifugation at 20,000×*g* (RT). The protein was precipitated from the supernatant by addition of ammonium sulfate to 40 % of saturation, followed by incubation on ice for 30 min. and centrifugation for 30 min. at 20,000×*g* (4 °C). The protein pellet was resuspended in Milli-Q water and precipitated using ammonium sulfate at 40 % of saturation as before. The pellet was then resuspended in Milli-Q water, desalted by extensive dialysis against Milli-Q water, and finally lyophilized.

### SDS-PAGE

SDS-PAGE was performed using the NuPAGE Novex System (ThermoFisher, Bleiswijk, The Netherlands) with 10 % Bis–Tris gels, MES SDS running buffer, and SeeBlue Plus2 pre-stained molecular mass markers. Prior to electrophoresis, all samples were heated for 10 min. at 70 °C in NuPAGE LDS Sample Buffer with NuPAGE Sample Reducing Agent, as per manufacturer’s recommendations for denaturing and reducing PAGE. Gels were stained using Coomassie SimplyBlue SafeStain (ThermoFisher, Bleiswijk, The Netherlands).

For detection of glycosylated proteins, SDS-PAGE gels were stained using Periodic acid-Schiff staining [33]. The gel was incubated for 1 h in 12.5 % TCA, 1 h in 1 % periodic acid/3 % acetic acid, 1 h in 15 % acetic acid (replaced every 10 min.), and 1 h at 4 °C in the dark in Schiff’s reagent (Sigma-Aldrich, Zwijndrecht, The Netherlands). The gel was then washed two times for 5 min. in 0.5 % sodium bisulfite and destained in 7 % acetic acid.

### Treatment of proteins with α-mannosidase or phosphatase

For α-mannosidase digestions, 30 µg of glycoprotein was incubated for 24 h at 37 °C under mild agitation with 0.9 U of jack bean α(1-2,1-3,1-6) mannosidase (Sigma-Aldrich, Zwijndrecht, The Netherlands) in 60 µL of 20 mM sodium acetate, 0.4 mM zinc chloride, pH 5. Dephosphorylation involved incubation of 30 µg of glycoprotein for 24 h at 37 °C under mild agitation with 60 U of calf intestinal alkaline phosphatase (NEB, Ipswich, MA) in 60 µL of 50 mM Tris–HCl, 10 mM magnesium chloride, pH 8.5.

For consecutive α-mannosidase/phosphatase digestions, the enzyme after each step was inactivated by heating for 15 min. at 100 °C, followed by desalting using Micro Bio-Spin P-6 columns (Bio-Rad, Veenendaal, The Netherlands). To allow mass spectrometric analysis of the reaction products, it was verified that such analysis of enzyme-only digestions revealed no significant peaks in the relevant ~38–41 kDa range (not shown).

### Mass spectrometry

Matrix-assisted laser desorption/ionization time-of-flight (MALDI-TOF) mass spectrometry was performed using an ultrafleXtreme mass spectrometer (Bruker, Leiderdorp, The Netherlands). Proteins were desalted using Micro Bio-Spin P-6 columns (Bio-Rad, Veenendaal, The Netherlands), and samples were prepared by the dried droplet method on a 600 µm AnchorChip target (Bruker, Leiderdorp, The Netherlands), using 5 mg mL^−1^ 2,5-dihydroxyacetophenone, 1.5 mg mL^−1^ diammonium hydrogen citrate, 25 % (v/v) ethanol and 3 % (v/v) trifluoroacetic acid as matrix. Spectra were derived from ten 500-shot (1000 Hz) acquisitions taken at non-overlapping locations across the sample. Wide mass-range measurements were made in the positive linear mode, with ion source 1, 25.0 kV; ion source 2, 23.3 kV; lens, 6.5 kV; pulsed ion extraction, 680 ns. Detailed analyses of glycoproteins in the ~38-41 kDa range were done with ion source 1, 20.0 kV; ion source 2, 18.4 kV; lens, 6.2 kV; pulsed ion extraction, 450 ns, and spectra were derived from ten 1000-shot (1000 Hz) acquisitions. Protein Calibration Standard II (Bruker, Leiderdorp, The Netherlands) was used for external calibration.

### Steady-state fluorescence spectroscopy

Steady-state fluorescence spectroscopy was performed on a Cary Eclipse Fluorescence Spectrophotometer (Agilent Technologies, Amstelveen, The Netherlands), monitoring the intrinsic fluorescence emission of the tryptophan residues in the WW domain at 340 nm, with excitation at 295 nm. Proteins were dissolved overnight in 10 mM sodium phosphate buffer (pH 7) at RT. The binding assays for both couples ${\mathbf{C}}_{{\mathbf{4}}}^{{\mathbf{P}}} - {\mathbf{D}}^{{{\mathbf{PPxY}}}}$/${\mathbf{C}}_{{\mathbf{4}}}^{{\mathbf{P}}} - {\mathbf{D}}^{{{\mathbf{WW}}}}$, and ${\mathbf{C}}_{{\mathbf{4}}}^{{\mathbf{P}}} - {\mathbf{D}}^{{{\mathbf{PPxY}} ^* }}$/${\mathbf{C}}_{{\mathbf{4}}}^{{\mathbf{P}}} - {\mathbf{D}}^{{{\mathbf{WW}}}}$ were conducted in triplicate at RT. A 500 μL aliquot of 10 μM ${\mathbf{C}}_{{\mathbf{4}}}^{{\mathbf{P}}} - {\mathbf{D}}^{{{\mathbf{WW}}}}$ was pipetted into a quartz fluorescence cuvette (Sigma-Aldrich, Zwijndrecht, The Netherlands). A 500 µM solution of ligand was stepwise added to the cuvette, up to a final ligand concentration of ~24 µM. The time interval between additions was 30 min. The final volume of added ligand solution did not exceed 5 % of the starting volume of ${\mathbf{C}}_{{\mathbf{4}}}^{{\mathbf{P}}} - {\mathbf{D}}^{{{\mathbf{WW}}}}$. Curve fitting for K_d_ determination was done on averaged data of triplicate titrations.

### Isothermal titration calorimetry

ITC was conducted on a MicroCal VP-ITC (Malvern Instruments, Malvern, United Kingdom) at 25 °C. All purified protein polymers were dissolved in 10 mM sodium phosphate buffer, pH 7 and filtrated with 0.2 μm Minisart NML Syringe Filters (Sigma-Aldrich, Zwijndrecht, The Netherlands). Prior to titration, each protein polymer solution was degassed under vacuum for 60 min. at RT. The ligand concentration of ${\mathbf{C}}_{{\mathbf{4}}}^{{\mathbf{P}}} - {\mathbf{D}}^{{{\mathbf{PPxY}}}}$ or ${\mathbf{C}}_{{\mathbf{4}}}^{{\mathbf{P}}} - {\mathbf{D}}^{{{\mathbf{PPxY}} ^* }}$ in the titration syringe was 2.9 mM. Each titration consisted of 63 injections at an interval of 250 s. Ligand aliquots of 4 µL were titrated into 1.4 mL of a 200 µM ${\mathbf{C}}_{{\mathbf{4}}}^{{\mathbf{P}}} - {\mathbf{D}}^{{{\mathbf{WW}}}}$ protein solution inside the calorimeter cell under continuous stirring at 329 rpm. Data obtained from the injection of ligand molecules into 1.4 mL of 10 mM PBS buffer (Additional file 1: Fig. S2) were subtracted as blanks from the experimental data before the data were analyzed using MicroCal Origin Software (Malvern Instruments, Malvern, United Kingdom). Titrations were performed in triplicate and K_d_ values were averaged after curve fitting.

## Additional file

10.1186/s12934-016-0498-3 This file consists of one supplemental table and two supplemental figures. **Table S1.** Oligonucleotides used in gene construction. **Figure S1.** MALDI-TOF of ${\mathbf{C}}_{{\mathbf{4}}}^{{\mathbf{P}}} - {\mathbf{D}}^{{{\mathbf{PPxY}}}}$ before and after phosphatase treatment. **Figure S2.** Control ITC measurements.

## Abbreviations

ITC: isothermal titration calorimetry
K_d_: dissociation constant
LDS: lithium dodecyl sulfate
MALDI-TOF: matrix-assisted laser desorption/ionization time-of-flight
Man: mannose
MES: 2-(N-Morpholino)ethanesulfonic acid
*m*/*z*: mass-to-charge ratio
OD_600_: optical density at 600 nm
P: phosphate
PAGE: polyacrylamide gel electrophoresis
PBS: phosphate-buffered saline
PCR: polymerase chain reaction
rpm: revolutions per minute
RT: room temperature
SDS: sodium dodecyl sulfate
TCA: trichloroacetic acid
Tris: tris(hydroxymethyl)aminomethane
