# Supplementary material for: Production in Pichia pastoris of complementary protein-based polymers with heterodimer-forming WW and PPxY domains
Source: Microb Cell Fact. 2016 Jun 10;15:105. doi: 10.1186/s12934-016-0498-3 (PMC4902918; doi:10.1186/s12934-016-0498-3)
Supplement: Supplementary file 1 — 10.1186/s12934-016-0498-3 This file consists of one supplemental table and two supplemental figures. Table S1. Oligonucleotides used in gene construction. Figure S1. MALDI-TOF of ${\mathbf{C}}_{{\mathbf{4}}}^{{\mathbf{P}}} - {\mathbf{D}}^{{{\mathbf{PPxY}}}}$C4P-DPPxY before and after phosphatase treatment. Figure S2. Control ITC measurements. [file 12934_2016_498_MOESM1_ESM.pdf]

Additional file 1

Production in *Pichia pastoris* of complementary protein-based polymers with heterodimer-forming WW and PPxY domains

Natalia E. Domeradzka[1,2†], Marc W.T. Werten[1†*], Renko de Vries[2], Frits A. de Wolf[1]

[†]These authors contributed equally to this work.

[1] Wageningen UR Food and Biobased Research, Bornse Weilanden 9, 6708 WG Wageningen, The Netherlands

[2] Physical Chemistry and Soft Matter, Wageningen University, Stippeneng 4, 6708 WE Wageningen, The Netherlands

* Corresponding author, mailing address:

Wageningen UR Food & Biobased Research
Bornse Weilanden 9
6708 WG Wageningen
The Netherlands

Phone : +31.317.483683
Fax : +31.317.475347

E-mail: marc.werten@wur.nl

Table S1. Oligonucleotides used in gene construction

Insert	Oligonucleotide Name	Oligonucleotide sequence
	WW-FW1	5’-GCGCTCGAGAAAAGAGAGGCTGAAGCTGGTCCACCCGGTGCTTTGCCTTCTGGTTGGGAA-3’
	WW-RV1	5’-GGTCAACGTAATAAGTACGACCGTGAGGATCCTTTCTTTGTTCCCAACCAGAAGGCAAAG-3’
**D^{WW}**	WW-FW2	5’-GTCGTACTTATTACGTTGACCATAACACTAGGACTACCACATGGGAAAGACCACAGCCATTGCCA-3’
	WW-RV2	5’-GTACGAATTCTATTAGCCACCGGCTGGTGCTCCAGGTGGCAATGGCTGTGGTCTT-3’
**D^{PPxY}**	PPxY-FW	5’-GCGCTCGAGAAAAGAGAGGCTGAAGCTGGTCCACCCGGTGCTGAATACCCTCCATACCCACCAC-3’
	PPxY-RV	5’-GTACGAATTCTATTAGCCACCGGCTGGACCAGATGGATAAGGAGGTGGTGGGTATGGAGGGTA-3’
**D^{PPxY*}**	PPxY*-FW	5’-GTGCTGAATACCCTCCATACCCACCACCTCCTTATCCAGCTGGTCCAGCCGGTGGCTAATAG-3’
	PPxY*-RV	5’-AATTCTATTAGCCACCGGCTGGACCAGCTGGATAAGGAGGTGGTGGGTATGGAGGGTATTCAGCACCGG-3’

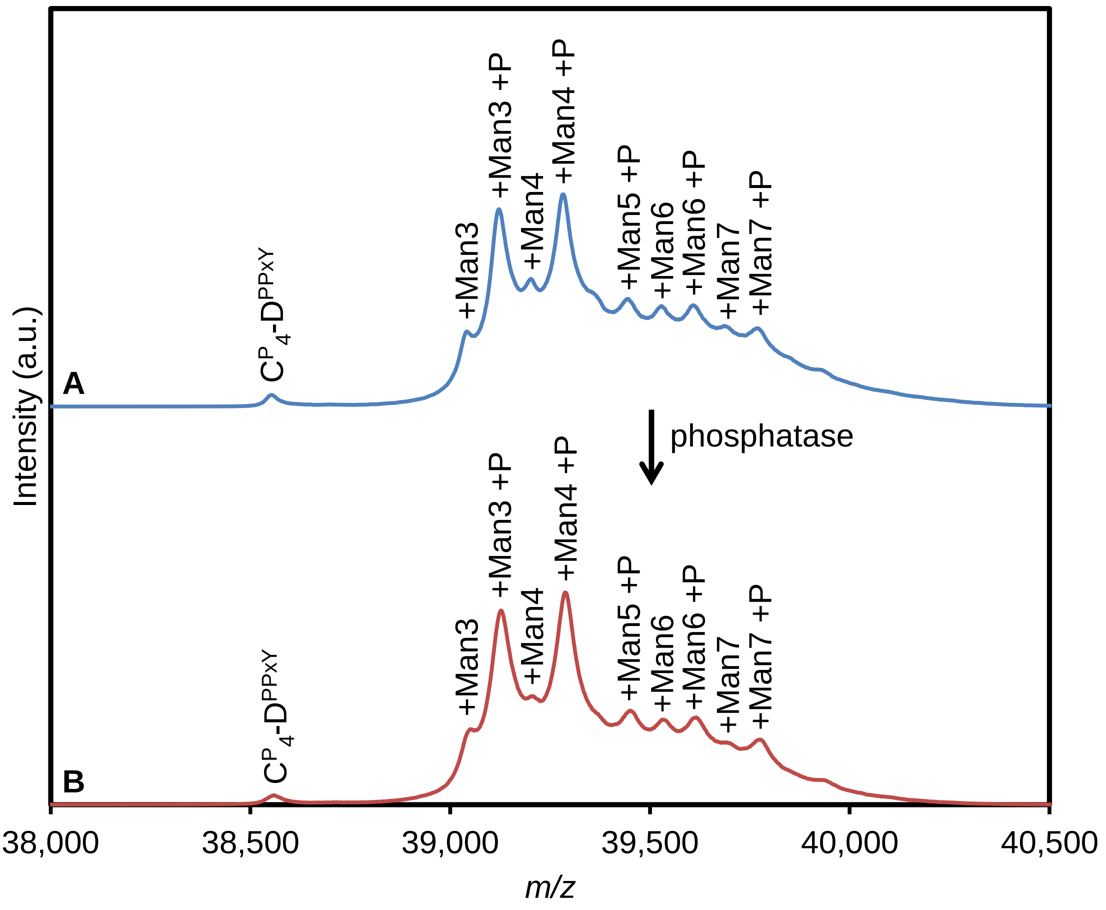

Fig. S1. MALDI-TOF of $C^P{}_4$-D^{PPxY} before (A) and after (B) phosphatase treatment. The same glycoforms are seen in both spectra.

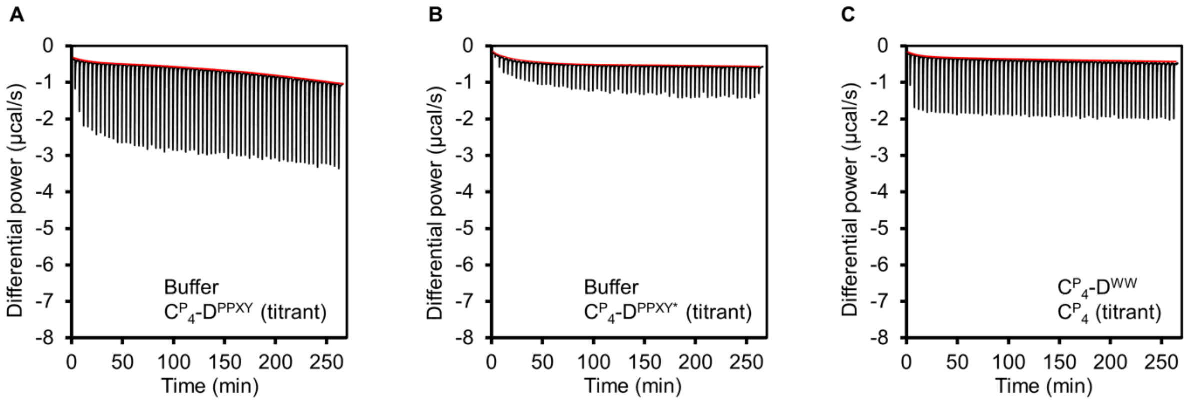

Fig. S2. Control ITC measurements. (A) Titration of buffer with $\mathbf{C^P{}_4\text{-}D^{PPxY}}$, (B) titration of buffer with $\mathbf{C^P{}_4\text{-}D^{PPxY*}}$, (C) titration of $\mathbf{C^P{}_4\text{-}D^{WW}}$ with $\mathbf{C^P{}_4}$ (protein without binding domain).
